# Supplementary material for: Oncological Outcomes of De-Escalation of Axillary Surgery in Breast Cancer Patients at a Referral Cancer Center in Colombia
Source: Cancers (Basel). 2025 Oct 22;17(21):3396. doi: 10.3390/cancers17213396 (PMC12610331; doi:10.3390/cancers17213396)
Supplement: Supplementary file 1 [file cancers-17-03396-s001.zip › cancers-3904957-supplementary.pdf]

cancers

MDPI

Supplementary material

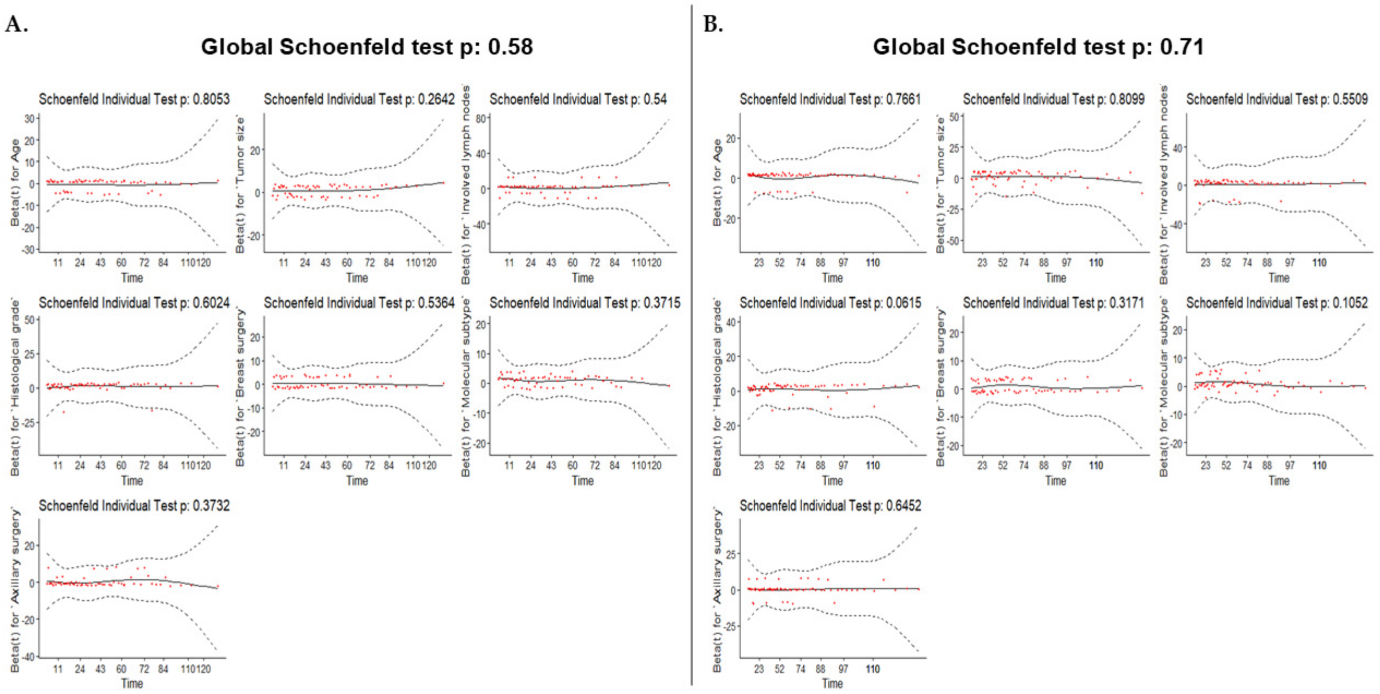

Figure S1. Analysis of the residuals of Shoenfeld: A) Time to recurrence. B) Overall survival

Supplementary material

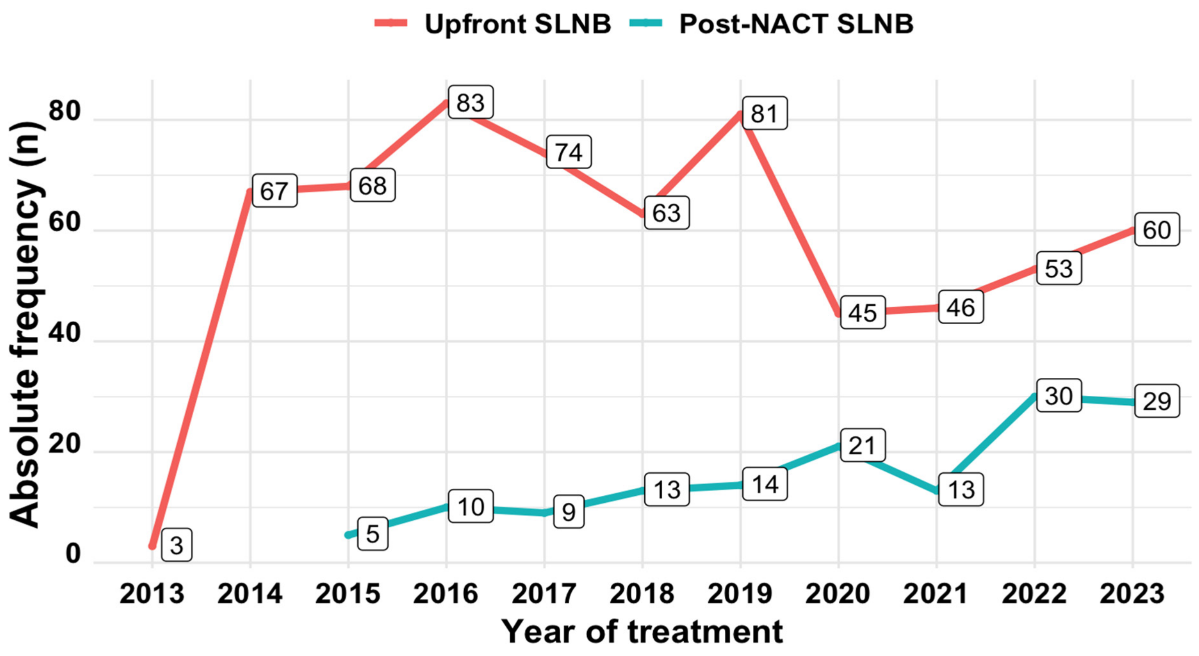

Figure S2. Number of procedures performed during the study period.
